# Modulating endothelial cell dynamics in fat grafting: the impact of DLL4 siRNA via adipose stem cell extracellular vesicles

**DOI:** 10.1152/ajpcell.00186.2024

**Published:** 2024-08-05

**Authors:** Sen-Lin Deng, Qiang Fu, Qing Liu, Fu-Jun Huang, Miao Zhang, Xun Zhou

**Affiliations:** ^1^Plastic and Aesthetic Department, People’s Hospital of Chongqing Banan District, Chongqing, People’s Republic of China; ^2^Department of Dermatology and Cosmetology, Chongqing Hospital of Traditional Chinese Medicine, Chongqing, People’s Republic of China; ^3^Banan District Center for Disease Control and Prevention, Chongqing, People’s Republic of China

**Keywords:** ADSC-derived extracellular vesicles, angiogenesis, autologous fat grafts, DLL4 siRNA, tissue engineering

## Abstract

In the context of improving the efficacy of autologous fat grafts (AFGs) in reconstructive surgery, this study delineates the novel use of adipose-derived mesenchymal stem cells (ADSCs) and their extracellular vesicles (EVs) as vehicles for delivering delta-like ligand 4 (DLL4) siRNA. The aim was to inhibit DLL4, a gene identified through transcriptome analysis as a critical player in the vascular endothelial cells of AFG tissues, thereby negatively affecting endothelial cell functions and graft survival through the Notch signaling pathway. By engineering ADSC EVs to carry DLL4 siRNA (ADSC EVs-siDLL4), the research demonstrated a marked improvement in endothelial cell proliferation, migration, and lumen formation, and enhanced angiogenesis in vivo, leading to a significant increase in the survival rate of AFGs. This approach presents a significant advancement in the field of tissue engineering and regenerative medicine, offering a potential method to overcome the limitations of current fat grafting techniques.

**NEW & NOTEWORTHY** This study introduces a groundbreaking method for enhancing autologous fat graft survival using adipose-derived stem cell extracellular vesicles (ADSC EVs) to deliver DLL4 siRNA. By targeting the delta-like ligand 4 (DLL4) gene, crucial in endothelial cell dynamics, this innovative approach significantly improves endothelial cell functions and angiogenesis, marking a substantial advancement in tissue engineering and regenerative medicine.

## Graphical abstract

**Figure:**
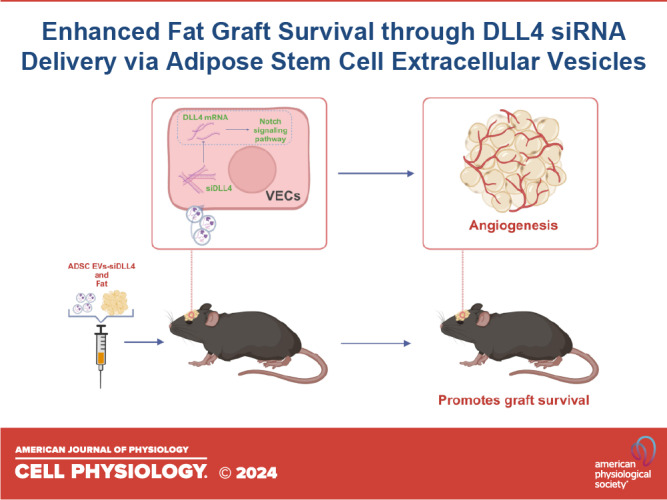

## INTRODUCTION

Fat graft has become a widely used technique in esthetic and reconstructive surgeries, demonstrating significant success (1–3). Among the factors contributing to its success is the survival of autologous fat graft (AFG), which is crucial (4). Adipose-derived stem cells (ADSCs), as an important cellular source, have shown potential for promoting fat survival (5–7). In addition, extracellular vesicles (EVs), which serve as an important means of cell communication, may play a vital role in ADSCs (8–10).

ADSCs are multifunctional stem cells with excellent proliferation and differentiation potential (11). In addition to direct effects, ADSCs can influence cell survival and tissue repair by releasing EVs (12, 13). EVs, as small extracellular vesicles, possess unique characteristics in terms of their morphology and molecular composition (14, 15). Through this mechanism, EVs play a crucial role in intercellular communication and the repair process (16–18). Delta-like ligand 4 (DLL4), as a regulator of the Notch signaling pathway, plays an important role in angiogenesis (19–21). It regulates the process of angiogenesis by inhibiting the proliferation and migration ability of vascular endothelial cells via the Notch signaling pathway (19, 21). The interaction between DLL4 and vascular endothelial cells is crucial for angiogenesis (22, 23). Thus, inhibiting DLL4 may be a strategy to promote angiogenesis and enhance AFG survival (24).

As a tool for gene silencing, siRNA has been widely applied in the field of gene silencing (25–27). Research has indicated that ADSC EVs have the potential to deliver siRNA (28). It has been found that ADSC EVs loaded with siDLL4 can promote the proliferation, migration, and lumen formation ability of microvascular endothelial cells (mVECs) (28). This suggests that ADSC EVs-siDLL4 has potential application value in promoting angiogenesis and enhancing AFG survival (29–31).

The purpose of this study is to identify genes related to AFG survival through high-throughput transcriptome sequencing (RNA sequencing, RNA-seq) and investigate the potential application of ADSC EVs loaded with siDLL4 in promoting angiogenesis and enhancing AFG survival. This research holds significant scientific importance as it aims to improve the clinical outcomes of AFGs and explore new possibilities for cell-based therapies in fat transplantation. ADSC EVs-siDLL4, as a potential biological therapeutic strategy, shows promising prospects for enhancing the effectiveness of AFG cell therapy and holds critical clinical application potential. Through further research and application, we hope to provide better options for fat transplantation therapy and improve patients’ quality of life.

## MATERIALS AND METHODS

### Animal Treatment

Seventy-two Female C57BL/6J mice (Strain Code: 219) at 6 wk of age were purchased from Beijing Vital River Laboratory Animal Technology Co., Ltd. (Beijing, PR China). The mice weighed 20 ± 2 g and were housed in standard cages under a 12-h light-dark cycle at a room temperature of 23 ± 1°C. They had ad libitum access to food and water. Before the start of the experiment, the mice underwent a 1-wk acclimation period. The experimental procedures and animal use protocol were approved by our institutional Animal Ethics Committee. All animal experiments in this study were conducted in accordance with internationally recognized animal welfare standards and relevant regulations. All feasible measures were taken to minimize animal pain and discomfort throughout the experiment. After the experiment, the animals were euthanized in a humane manner.

For the fat grafting procedure, the scalp area of the mice was selected as it had no subcutaneous fat. The mice were anesthetized using isoflurane (R510-22-10, RWD, Shandong, PR China). Subcutaneous fat tissue from the inguinal region of donor mice was harvested, cut into small pieces, and then inserted into a subcutaneous tunnel in the scalp of recipient mice using a 16-G needle. The needle was subsequently withdrawn, and fat fragments (200 μL) or a mixture were injected retrogradely into the tunnel, followed by wound closure (32). Twelve weeks after transplantation, micro-computed tomography (micro-CT) using a SkyScan 1275 machine (Bruker) was performed to measure the volume of the transplant, which was then harvested. The weight of the graft was determined using a scale, and the volume was measured using the fluid displacement method. In brief, PBS was added to a graduated cylinder, and the transplant was fully immersed in PBS. The increase in solution volume determined the volume of the transplant (33).

For the RNA-seq experiment, six mice were randomly divided into two groups, with *n* = 3/group. One group served as the subcutaneous fat (SF) control group, and the other group as the adipose-derived fat grafting (AFG) group. The SF group received subcutaneous fat tissue harvested from the inguinal region directly, whereas the AFG group underwent fat grafting, and the transplants were harvested 12 wk after transplantation for RNA-seq.

For the experiment to validate the expression of DLL4 in the grafted fat tissue, 12 mice were randomly divided into two groups, with *n* = 6/group. One group served as the subcutaneous fat control group (SF), and the other group as the adipose-derived fat grafting (AFG) group. The SF group received subcutaneous fat tissue harvested from the inguinal region directly, whereas the AFG group underwent fat grafting, and the transplants were harvested 12 wk after transplantation for subsequent experiments.

For the experiment investigating the effects of anti-DLL4 antibody on transplant survival, 18 mice were randomly divided into three groups, with *n* = 6/group. The groups were as follows: saline control group (Mock), isotype control group, and anti-DLL4 antibody treatment group. All groups underwent fat grafting, and on the day of fat transplantation, they received intraperitoneal injections of saline (100 μL), isotype control (Armenian hamster IgG, 5 mg/kg) (BE0091, Bio X Cell), or anti-DLL4 Ab (Armenian hamster anti-mouse, 5 mg/kg) (BE0127, Bio X Cell). The mice were maintained according to standard housing conditions, and relevant indicators were measured at the end of the experiment (24).

For the experiment to assess the effects of ADSC EVs-siDLL4 on transplant survival in vivo, 24 mice were randomly divided into four groups, with *n* = 6/group. The groups were siRNA negative control (siNC), siRNA for DLL4 (siDLL4), ADSC EVs, and ADSC EVs-siDLL4. siNC, siDLL4, ADSC EVs, and ADSC EVs-siDLL4 were mixed with fat fragments (10 μg) separately and then transplanted into the subcutaneous region of the mice’s heads (200 μL). siNC and siDLL4 were delivered using liposome carriers. The mice were maintained according to standard housing conditions, and relevant indicators were measured at the end of the experiment (33). In the experimental procedure involving isolation and in vitro culture of mouse vascular endothelial cells, a total of 12 mice were used. The euthanasia of all animals was conducted via CO_2_ asphyxiation followed by cervical dislocation to ensure death, in accordance with the guidelines of our animal ethics committee. This approach was implemented to minimize animal suffering and distress to the greatest extent possible.

### RNA-Seq and Data Quality Control

Total RNA from each sample was extracted using Trizol reagent (16096020, Thermo Fisher). The concentration, purity, and integrity of RNA were assessed using the Qubit2.0 Fluorometer (Life Technologies, Q33216) with the Qubit RNA Analysis Kit (HKR2106-01, Shanghai Baoji Biotechnology Co., Ltd., Shanghai, PR China), Nanometer UV-Vis spectrophotometer (IMPLEN), and RNA Nano 6000 Assay Kit on the Bioanalyzer 2100 system (Agilent, 5067-1511). The total RNA concentration for each sample was 3 μg, which was used as the input material for RNA sample preparation.

Following the manufacturer’s recommendations, the NEBNext UltraTM RNA Library Preparation Kit (NEB, E7435L, Beijing, PR China) compatible with Illumina (USA) was used to generate cDNA libraries, and their quality was evaluated on the Agilent Bioanalyzer 2100 system. Index-coded samples were then clustered on the cBot cluster generation system (Illumina) using the TruSeq PE Cluster Kit v3 cBot HS (Illumina) (PE-401-3001, Illumina), as per the manufacturer’s instructions. The libraries were sequenced on the Illumina Hiseq 550 platform, generating 125-bp/150-bp paired-end reads.

The quality of the raw sequencing data’s paired-end reads was assessed using FastQC software v0.11.8. The raw data were preprocessed using Cutadapt software 1.18 to remove Illumina sequencing adapters and poly(A) tails. Perl scripts were used to discard reads with more than 5% N content. The FASTX Toolkit software 0.0.13 was used to extract reads with a base quality above 20, accounting for 70% of the bases. The BBMap software was used to repair the paired-end sequences. Finally, the filtered high-quality read fragments were aligned to the mouse reference genome using HISAT2 software (0.7.12) (34–37).

### Bioinformatics Analysis

RNA-seq data were analyzed for differentially expressed genes (DEGs) using the “limma” package in R software, and adjusted *P* values (adj*P*) were calculated using the Benjamini–Hochberg (BH) method. DEGs were selected based on the criteria of |log2FC| > 2 and adj*P* < 0.05. The volcano plot and heatmap of DEGs were generated using the “pheatmap” package in R software.

A gene set related to “fat grafts” was retrieved from the GeneCards database (https://www.genecards.org/) and intersected using the “VennDiagram” package in R software. The intersected genes were further subjected to Gene Ontology (GO) and Kyoto Encyclopedia of Genes and Genomes (KEGG) enrichment analysis using the “clusterProfiler” package in R software. The enrichment results were visualized as bar plots using the “ggplot2” package. In addition, the protein-protein interaction (PPI) network of the intersected genes was constructed using the STRING database (https://cn.string-db.org/).

### Isolation and In Vitro Cultivation of Mouse Endothelial Cells

mVECs were isolated and used for in vitro experiments. The following methods were used: first, mice were euthanized, and the abdominal aorta was dissected and immediately immersed in ice-cold Hank’s balanced salt solution (HBSS) (14175095, Thermo Fisher). Subsequently, under a dissecting microscope, the adipose tissue and connective tissue on the adventitia were carefully removed, and the vessel lumen was flushed with HBSS to eliminate blood. Next, the vessel wall was longitudinally cut and placed endothelial-side down in Dulbecco’s modified Eagle medium-Ham’s F-12 (DMEM-H) (11965092, Thermo Fisher) containing 0.5% Trypsin-EDTA (15400054, Thermo Fisher) at 37°C for 15 min. Digestion was terminated by adding 20% fetal bovine serum (FBS) (10100147C, Thermo Fisher). The vessel wall was then transferred to another culture dish containing DMEM-H and gently scraped with a surgical blade to collect the endothelial cells. The cells were centrifuged at 1,000 *g* for 10 min, the supernatant was removed, and the cell concentration was adjusted to 1.5 × 10^5^ cells/mL. The cells were seeded onto gelatin-coated dishes in DMEM-H supplemented with 10% FBS and 1% penicillin-streptomycin (P-S) (15140163, Thermo Fisher) and incubated at 37°C with 5% CO_2_. After 24 h, nonadherent cells were removed, and a fresh culture medium was added for further cultivation (38). The isolated mVECs were identified using Alexa Fluor 488 anti-CD31 antibody (rat anti-mouse, 1:50) (ab307133, Abcam, UK) for immunofluorescence staining, with a purity of over 90%.

### Cell Transfection and Treatment

mVECs overexpressing and knockdown of DLL4 were constructed using lentivirus-mediated transfection. Plasmids encoding the target gene and helper plasmids were cotransfected into 293T cells (CRL-3216, ATCC) provided by Shanghai Biotechnology (PR China) for lentivirus packaging services. The packaged lentivirus was obtained after validation, amplification, and purification. For lentivirus-mediated cell transfection, 5 × 10^5^ cells were seeded in a 6-well plate. When the cell confluence reached 70–90%, the medium containing an appropriate amount of packaged lentivirus (multiplicity of infection, MOI = 10, working titer ∼5 × 10^6^ TU/mL) and 5 μg/mL of polybrene (Merck, TR-1003) was added for transfection. After 4 h, an equal amount of medium was added to dilute the polybrene, and the fresh medium was replaced 24 h later. The transfection efficiency was observed through luciferase reporter gene expression 48 h later, and subsequent stable cell lines were obtained by using 1 μg/mL of puromycin (A1113803, Thermo Fisher) for resistance selection (39). For the knockdown cell lines, two different shRNA sequences were used, and the one with higher efficiency was selected for further experiments, as shown in Table 1.

**Table 1.** shRNA and siRNA sequences

Name	sh-RNA Sequences (5′–3′)
sh-NC/siNC	CCTAAGGTTAAGTCGCCCTCG
sh-DLL4-1 (mouse)	GCCGGACTTTCTTCCGCATTT
sh-DLL4-2 (mouse)	GCAGAACCACACATTGGACTA
siDLL4-1 (mouse)	GGGCAUCUGUAUUUCCAAA
siDLL4-2 (mouse)	GCCUGUGCAAUGAAUGUAU

DLL4, delta-like ligand 4; NC, negative control.

siNC and siDLL4 were transfected using the lipofectamine-mediated method, and the knockdown efficiency of siDLL4 was validated through subsequent experiments. Cells (5 × 10^5^) were seeded in a 6-well plate. When the cell confluence reached 70–90%, transfection was performed using Lipofectamine 3000 (L3000150, Thermo Fisher) according to the manufacturer’s protocol. After 72 h of transfection, the cells were assessed for DLL4 knockdown by RT-qPCR and Western blot. The siRNA with a better knockdown effect was selected for loading into ADSC EVs.

For the experiments investigating changes in mVECs proliferation, migration, and angiogenesis, isotype control (Armenian hamster IgG, 5 μg/mL) (BE0091, Bio X Cell), anti-DLL4 antibody (Armenian hamster anti-mouse, 5 μg/mL) (BE0127, Bio X Cell), BMS-986115 (20 nM) (HY-12860, MCE), siNC (5 μg/mL), siDLL4 (5 μg/mL), ADSC EVs (5 μg/mL), or ADSC EVs-siDLL4 (5 μg/mL) were added to the corresponding culture medium based on the experimental requirements.

### CCK-8 Assay for Cell Proliferation

Cell viability and proliferation were assessed using the cell counting kit (CCK)-8 assay kit (C0037, Beyotime, Shanghai, PR China). Cells in the logarithmic growth phase were adjusted to a concentration of 5 × 10^4^ cells/mL in DMEM-H medium supplemented with 10% FBS. The cell suspension was added to a 96-well culture plate, with 100 μL/well, and incubated in a cell culture incubator. After 24, 48, and 72 h, the absorbance of the cells was measured. For the CCK-8 assay, 10 μL of the CCK-8 working solution was added to each well, followed by incubation at 37°C for 2 h. The absorbance at 450 nm was measured using a Multiskan FC microplate reader (51119080, Thermo Fisher). Three replicate wells were set up for each group, and the average value was calculated. The experiment was repeated three times.

### Scratch Assay for Cell Migration

mVECs were seeded and cultured in a 6-well plate until confluence was reached. A 1,000-μL pipette tip was used to create a scratch perpendicular to the cell plane, followed by washing with PBS to remove cell debris. After 12 h of incubation, cell migration was evaluated using an inverted microscope (IX73, Olympus, Japan), and the results were recorded. Cell migration was analyzed using Image-Pro Plus 6.0 (Media Cybernetics), where the migration rate (%) was calculated as the migration area divided by the initial scratch area multiplied by 100% (40). The experiment was repeated three times.

### In Vitro Tubule Formation Experiment

Matrigel (356234, Corning) was thawed at 4°C and uniformly mixed before being placed on a prechilled 96-well plate (50 μL/well). The plate was then incubated at 37°C to allow the gel to solidify. Subsequently, mVECs were seeded into the Matrigel-coated wells (2 × 10^4^ cells/well), and after 12 h of incubation, the results were observed and recorded using a differential interference microscope (IX73, Olympus, Japan). The length of the tubules was quantitatively analyzed using Image-Pro Plus 6.0 (Media Cybernetics) (41). The experiment was repeated three times.

### Culture of ADSCs and Isolation of EVs

Mouse ADSCs (CP-M138, Procell, Wuhan, PR China) were cultured in Mouse Adipose-Derived Mesenchymal Stem Cell Growth Medium (CM-M138, Procell, Wuhan, PR China). Prior to EV isolation, logarithmic phase ADSCs were seeded into a complete medium without EVs and incubated in a cell culture incubator at 37°C and 5% CO_2_. After 48 h of culture, the supernatant was collected, filtered through a 0.22-μm filter to remove cell aggregates, and then centrifuged at 300 *g* for 10 min at 4°C to remove cells. Subsequently, centrifugation at 3,000 *g* for 10 min was performed to eliminate dead cells, followed by centrifugation at 10,000 *g* for 30 min to remove cell debris, resulting in a cell debris-free conditioned medium (CM). Next, centrifugation at 110,000 *g* for 40 min was carried out; the bottom liquid in the centrifuge tube was retained, and the pellet was resuspended. This suspension was then subjected to centrifugation at 110,000 *g* for 90 min at 4°C to obtain the pellet, which was resuspended in 10 mL of PBS. Subsequently, another centrifugation at 110,000 *g* for 90 min at 4°C was performed, and the resulting pellet was considered as the EVs. The EVs were resuspended in 100 μL of PBS and stored at −80°C for further use. The obtained EVs were quantified for protein content using the bicinchoninic acid (BCA) assay kit (P0012S, Beyotime, PR China) (42).

### Electroporation for the Construction of ADSC EVs-siDLL4

ADSC EVs were used as the raw material for the synthesis of ADSC EVs-siDLL4 using electroporation. In a dish with a 0.1 cm electrode gap, ADSC EVs with a total protein concentration of 20 μg and siDLL4 at 20 μg were electroporated. The final volume of the electroporation sample was 400 μL, with PBS added to the mixture. A total of eight pulses were applied with an interval of 5 ms between each pulse and a voltage of 750 V. Subsequently, the sample was mixed with serum-free Mouse Adipose-Derived Mesenchymal Stem Cell Growth Medium at 37°C and incubated for 30 min on a rotator. The sample was then further incubated overnight at 4°C. Unbound siDLL4 and larger fragments were removed using an Optima XPN-100 differential ultracentrifuge. Larger fragments were cleared by centrifuging at 1,000 rpm for 5 min (5810 R), whereas smaller fragments were removed by centrifuging at 15,000 rpm for 20 min (SW32Ti, Beckman Coulter). Finally, ADSC EVs-siDLL4 was formed into spherical shapes by centrifuging at 31,000 rpm for 114 min using a centrifuge (SW32Ti, Beckman Coulter). ADSC EVs-siDLL4 was resuspended in PBS and stored at −80°C for further use.

### Characterization of ADSC EVs and ADSC EVs-siDLL4

Samples of ADSC EVs and ADSC EVs-siDLL4 were collected and protein was extracted. Western blot was performed to detect the expression of the positive markers CD9 and CD63, and the expression of the negative marker Calnexin. For detailed steps and antibody information, please refer to the description of the Western blot method.

#### Transmission electron microscopy.

ADSC EVs and ADSC EVs-siDLL4 were fixed immediately after precipitation in 2.5% glutaraldehyde at 4°C. Following fixation, the samples were dehydrated in a gradient alcohol series and embedded in epoxy resin. The ultrathin sections were stained with uranyl acetate and lead citrate and observed under a transmission electron microscope (JEM-1010, JEOL, Japan). Each experiment was repeated three times.

#### Nanoparticle tracking analysis.

The obtained ADSC EVs and ADSC EVs-siDLL4 were diluted in PBS to a particle concentration of 10^6^/mL–10^9^/mL, and then an appropriate amount of sample was added to the Nanosight analyzer (Nanosight NS300, Malvern Panalytical, UK) for detection and analysis (39). The experiment was repeated three times.

### Analysis of EVs Uptake/Internalization

ADSC EVs and ADSC EVs-siDLL4 were labeled using the PKH26 red fluorescent cell linker kit (PKH26GL, Merck) according to the manufacturer’s instructions. The labeled EVs were re-suspended in culture medium and added to mVECs, followed by incubation at 37°C and 5% CO_2_ for 2 h. After being washed three times with PBS, the cells were fixed with 4% paraformaldehyde for 30 min and co-stained with DAPI to visualize the cell nuclei. The experimental results were observed and saved using a laser confocal fluorescence microscope (STELLARIS 5, Leica, Germany), and the uptake rate of EVs (PKH26 positivity) was analyzed using a flow cytometer (BD LSRFortessa, BD Bioscience) (43).

### Immunofluorescence

Tissue samples were fixed with 4% paraformaldehyde, dehydrated in a sucrose gradient, and embedded in optimal cutting temperature (OCT) to produce 20-μm thick slices. Before the incubation with primary antibodies, the slices were rehydrated and blocked with 2% BSA. The appropriate primary antibodies were incubated overnight at 4°C, followed by the addition of corresponding fluorescent secondary antibodies for 1.5 h at room temperature. Excess secondary antibodies were removed by washing, and if necessary, counterstaining was performed with DAPI (62248, Thermo Fisher) for 10 min, followed by sealing of slides with an antiquenching mounting medium (44). Staining results were observed and saved using a laser confocal fluorescence microscope (STELLARIS 5, Leica, Germany). Semiquantitative analysis of staining results was performed using Image-Pro Plus 6.0 (Media Cybernetics) (45).

In the assessment of vascular DLL4 expression, the DLL4 expression rate (%) was calculated as DLL4-positive area divided by CD31-positive area multiplied by 100%. In the evaluation of tissue necrosis area, necrosis area (%) was calculated as Perilipin-negative area divided by total tissue area multiplied by 100%. In the evaluation of tissue angiogenesis, the vascular area is referred to as the CD31-positive area.

The primary antibodies used in this experiment included anti-DLL4 Ab (rabbit anti-mouse, 1:100) (PA5-85931, Thermo Fisher), anti-CD31 Ab (mouse anti-mouse, 1:50) (ab7388, Abcam, UK), and anti-Perilipin Ab (rabbit anti-mouse, 1:100) (ab108323, Abcam, UK).

The fluorescent secondary antibodies used in this experiment included: goat anti-rabbit IgG H&L (Alexa Fluor 488) (1:1,000) (ab150077, Abcam, UK), goat anti-rat IgG H&L (Alexa Fluor 647) preadsorbed (1:1,000) (ab150167, Abcam, UK), goat anti-rat IgG H&L (Alexa Fluor 488) (1:1,000) (ab150157, Abcam, UK), and goat anti-rabbit IgG H&L (Alexa Fluor 405) (1:1,000) (ab175652, Abcam, UK).

### Vascular Perfusion Analysis

After anesthetizing the mice, 100 μL of DyLight 488-Lycopersicon esculentum (Tomato) Lectin (1 mg/mL) (L32470, Thermo Fisher) was administered intravenously before euthanasia. After 30 min, the circulatory lectin was removed by perfusing PBS through the heart. Immunofluorescence analysis was conducted on harvested and processed tissue sections using an anti-CD31 antibody (rabbit anti-mouse, 1:100) (ab222783, Abcam, UK) to stain the tissue vasculature. Vascular perfusion was observed and documented using a laser scanning confocal microscope (STELLARIS 5, Leica, Germany). The results were quantitatively analyzed using Image-Pro Plus 6.0 (Media Cybernetics), with the vascular perfusion area (%) calculated as lectin-positive area divided by CD31-positive area, multiplied by 100% (24).

### Western Blot

Sample protein extraction was performed using the protein extraction kit (BB3101, BestBio, Shanghai, PR China), and the protein concentration was determined using the BCA kit (P0012S, BestBio, Shanghai, PR China). A 10% SDS-PAGE gel (P0012A, BestBio, Shanghai, PR China) was prepared, and each well was loaded with 50 μg of protein sample. The gel was run at a constant voltage of 80 V until stable and then switched to 120 V for a 2-h duration. Electrophoresis was carried out for 90 min, maintaining a constant current of 250 mA. Protein transfer to a PVDF membrane (IPVH00010, Merck, Germany) was performed. The PVDF membrane was blocked with Tris-buffered saline-Tween 20 (TBST) solution containing 5% skim milk at room temperature for 2 h. The blocking solution was removed, and the membrane was washed with TBST for 10 min, repeating the wash step three times. The membrane was incubated overnight at 4°C with the primary antibody (see Table 2 for antibody information), followed by three washes with TBST for 10 min each. Goat anti-rabbit IgG (1:2,000) (ab6721, Abcam, UK) or goat anti-mouse IgG (1:2,000) (Abcam, ab6789, UK) was used to incubate the membrane at room temperature for 1 h, followed by three washes with phosphate-buffered saline with Tween-20 (PBST) for 10 min each. The membrane was exposed to an ECL reaction solution (P0018FS, BestBio, Shanghai, PR China) for visualization and then exposed and developed in a darkroom (46). Each sample experiment was repeated three times.

**Table 2.** Western blot antibody information

Targets (Host and Reactivity)	Manufacturer	Cat. No.	Tested Dilution
DLL4 (rabbit anti-mouse)	Thermo Fisher	PA5-85931	1:500
N Cadherin (rabbit anti-mouse)	Abcam	ab76011	1:5,000
E Cadherin (mouse anti-mouse)	Abcam	ab231303	1:1,000
VEGF (rabbit anti-mouse)	Abcam	ab32152	1:1,000
HES1 (rabbit anti-mouse)	Abcam	ab71559	1:1,000
HEY1 (rabbit anti-mouse)	Abcam	ab235173	1:1,000
CD9 (mouse anti-mouse)	Thermo Fisher	10626D	1:1,000
CD63 (rabbit anti-mouse)	Abcam	ab216130	1:100
Calnexin (rabbit anti-mouse)	Abcam	ab22595	1:1,000
GAPDH (mouse anti-mouse)	Abcam	ab8245	1:500
β-actin (mouse anti-mouse)	Abcam	ab6276	1:5,000

Abcam, UK; Thermo Fisher. DLL4, delta-like ligand 4.

### Detection of Gene Expression by RT-qPCR

Total RNA was extracted from the samples using Trizol (16096020, Thermo Fisher). Subsequently, the One Step TB Green PrimeScript RT-PCR Kit (RR066A, Takara, Japan) was used to prepare the reaction mixture for reverse transcription and PCR (one-step method). RT-qPCR reactions were performed on a Thermal Cycler Dice Real-Time System III (TP990, Takara, Japan). The program consisted of a reverse transcription stage (42°C for 5 min, 95°C for 10 s, number of cycles = 1), a PCR stage (95°C for 5 s, 60°C for 34 s, number of cycles = 40), and a melting curve stage (95°C for 15 s, 60°C for 1 min, 95°C for 15 s, number of cycles = 1). Amplification and melting curves were used to verify the results. The fold change in target gene expression between the experimental and control groups was calculated using the 2^−ΔΔCt^ method, where ΔΔCT = ΔCt_test_ − ΔCt_control_, and ΔCt = Ct_target_ − Ct_reference_. Ct refers to the number of amplification cycles required for the real-time fluorescence intensity to reach the set threshold (44). Each sample was set up in triplicate, and the experiment was repeated three times. The primer sequences can be found in Table 3, with GAPDH serving as the internal reference gene.

**Table 3.** RT-qPCR primer sequences

Gene Names (Species)	Sequences (5′–3′)
*DLL4* (mouse)	F: TTCCAGGCAACCTTCTCCGA
	R: ACTGCCGCTATTCTTGTCCC
*GAPDH* (mouse)	F: AGGTCGGTGTGAACGGATTTG
	R: TGTAGACCATGTAGTTGAGGTCA

DLL4, delta-like ligand 4; F, forward; R, reverse.

### Statistical Analysis Methods

The statistical analysis of bioinformatics results was conducted using R 4.3.0, whereas other results were analyzed using SPSS 26.0 (IBM). Continuous data are presented as means ± standard deviation. For the analysis of data differences, normality and homogeneity of variance were assessed. If the data followed a normal distribution and exhibited homogeneity of variance, an independent samples *t* test was performed; otherwise, a nonparametric test was used. A significance level of *P* < 0.05 was considered statistically significant.

## RESULTS

### Key Role of DLL4 in Increased Graft Survival in AFG

AFG is a promising technique, but various common issues often lead to low graft retention. Therefore, identifying targets that can improve graft survival rate is crucial (47).

**Figure 1.**
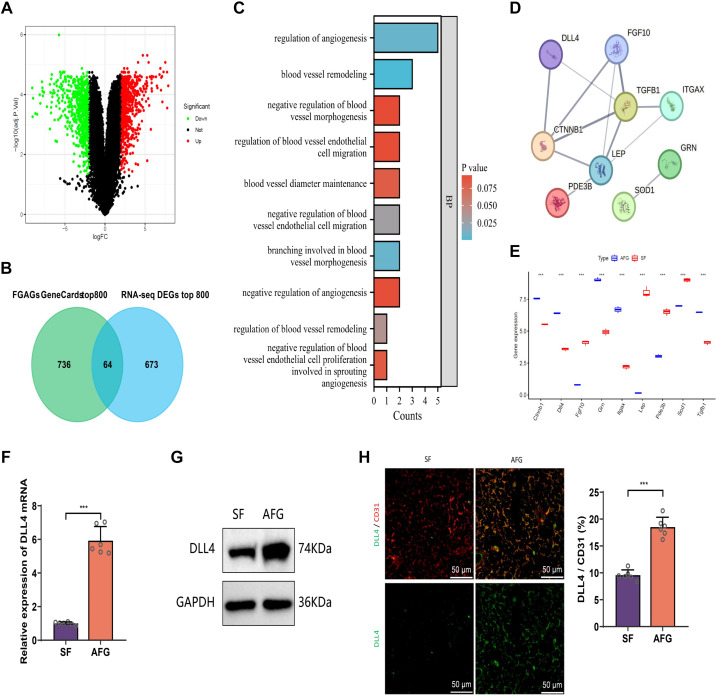
Delta-like ligand 4 (DLL4) exhibits high expression in autologous fat graft (AFG) tissue. *A*: volcano plot of differentially expressed genes (DEGs) analysis results from RNA-seq data, where red dots represent upregulated genes and green dots represent downregulated genes (*n* = 3 for each group). *B*: intersection of top 800 DEGs from RNA-sequencing (RNA-seq) and top 800 fat graft-related genes from GeneCards. *C*: enriched GO terms related to angiogenesis from the intersection of 64 genes. *D*: PPI network of genes involved in the 10 GO terms, with an interaction score of 0.4. *E*: expression profiles of nine angiogenesis-related genes in the RNA-seq data. *F* and *G*: expression of DLL4 in transplants harvested 12 wk after AFG as detected by RT-qPCR and Western blot, with subcutaneous fat (SF) from the donor site as the control (*n* = 6 for each group). *H*: expression of DLL4 and vascular marker CD31 in AFG transplant tissues detected by immunofluorescence, with representative images on the left and quantification of DLL4-positive vessels (CD31-positive) on the right (scale bar = 100 μm, *n* = 6 for each group); GO, Gene Ontology; PPI, protein-protein interacation. ***P < 0.001.

**Figure 2.**
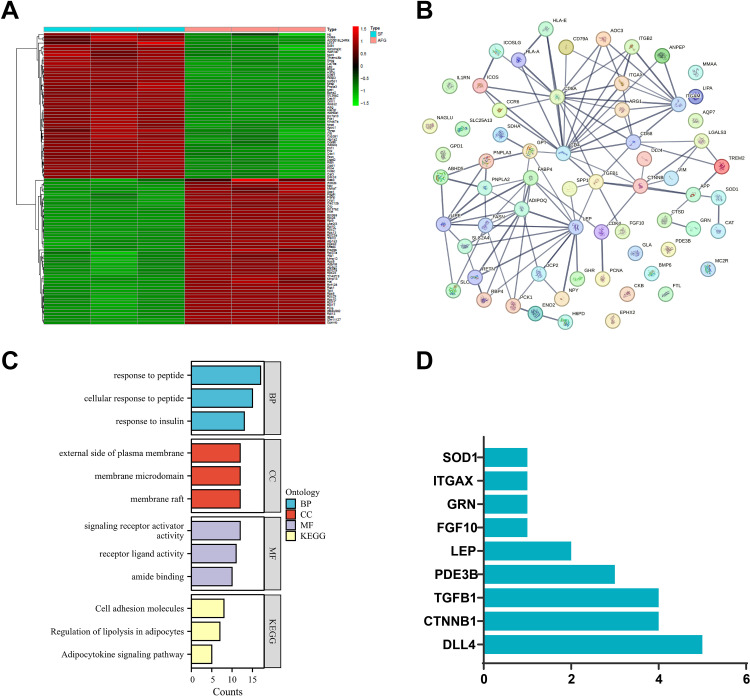
Heatmap of RNA-seq differentially expressed genes (DEGs), protein-protein interaction (PPI) network of intersecting genes, and functional enrichment analysis results. *A*: heatmap displaying the top 50 genes (ranked by adj*P*) from the DEGs analysis of RNA-sequencing (RNA-seq) data. *B*: PPI network of the intersecting candidate genes obtained from the STRING database, with an interaction score of 0.7. *C*: Gene Ontology (GO) and Kyoto Encyclopedia of Genes and Genomes (KEGG) enrichment analysis results for the 64 intersecting candidate genes. *D*: frequency statistics of genes involved in the 10 vascular-related GO terms.

To explore the key factors influencing AFG survival rate, we constructed an AFG model using subcutaneous fat tissue in mice. After 12 wk posttransplantation, we harvested the transplanted tissues (AFG) and performed RNA-seq, comparing them to the control SF tissue. Following data quality control and filtering, we selected DEGs between groups based on the criteria |logFC| > 2 and adj*P* < 0.05 (RNA-seq DEGs). The results showed that there were 1,404 DEGs (RNA-seq DEGs) between SF and AFG, with 719 upregulated and 685 downregulated genes in AFG (Figs. 1*A* and 2*A*).

Subsequently, we retrieved 2,650 fat graft-associated genes (FGAGs) from the GeneCards database (https://www.genecards.org/) using the keyword “fat grafts.” We then intersected the top 800 FGAGs (ranked by Relevance score) with the top 800 RNA-seq DEGs (ranked by adj*P*). To align the analysis closer to the human species, we converted the 800 mouse genes into their corresponding human gene names, resulting in 737 genes for intersection. Ultimately, we obtained 64 candidate genes closely related to AFG (Figs. 1*B* and 2*B*). Functional enrichment analysis indicated that these 64 candidate genes were primarily involved in GO terms such as response to peptide, cellular response to peptide, response to insulin, and KEGG signaling pathways such as regulation of lipolysis in adipocytes, cell adhesion molecules, and adipocytokine signaling pathway (Fig. 2*C*).

Post-graft angiogenesis is a critical factor affecting graft survival (47). Further analysis of the functional enrichment results (GO and KEGG) revealed that 35 candidate genes were enriched in 10 GO terms related to angiogenesis (Fig. 1*C*). These genes included CTNNB1, DLL4, FGF10, GRN, ITGAX, LEP, PDE3B, SOD1, and TGFB1 (Fig. 1*D*). Among them, CTNNB1, DLL4, GRN, ITGAX, and TGFB1 were upregulated in AFG, whereas FGF10, LEP, PDE3B, and SOD1 were downregulated in AFG (Fig. 1*E*). By counting the frequencies of these nine genes appearing in the 10 angiogenesis-related GO terms, we found that DLL4 had a higher occurrence rate of 5 (Fig. 2*D*). DLL4 negatively regulates angiogenesis, and its high expression has an inhibitory effect on AFG survival. Therefore, we hypothesize that intervening in the expression of DLL4 in AFG tissues is crucial for promoting graft survival.

To validate the aforementioned hypothesis, we conducted validation experiments on the expression of DLL4 in mouse AFG (12 wk posttransplantation) and SF tissues. Results from RT-qPCR, Western blot, and immunofluorescence staining showed that DLL4 was significantly upregulated in AFG compared with SF (Fig. 1, *F–H*). In addition, immunofluorescence staining demonstrated that DLL4 was colocalized with vascular markers in AFG tissue, indicating that DLL4 is mainly expressed in endothelial cells (Fig. 1*H*).

In summary, both the analysis and experimental results indicate that DLL4 is upregulated in AFG and primarily localizes to endothelial cells.

### DLL4 Inhibition Promotes Angiogenic Potential of Endothelial Cells

**Figure 3.**
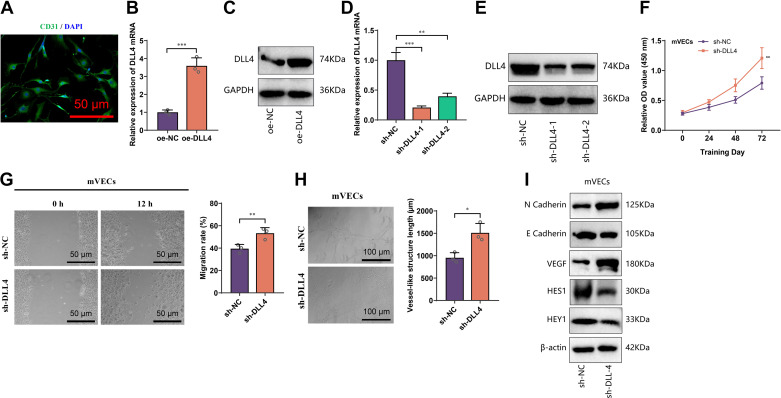
Overexpression and knockdown of delta-like ligand 4 (DLL4) in microvascular endothelial cells (mVECs) and the effect of DLL4 knockdown on vascular formation ability in mVECs. *A*: representative immunofluorescence images showing CD31 expression in primary mVECs (scale bar = 25 μm). *B* and *C*: RT-qPCR and Western blot analysis confirming DLL4 overexpression in mVECs. *D* and *E*: RT-qPCR and Western blot analysis confirming DLL4 knockdown in mVECs, with sh-DLL4-1 (referred to as sh-DLL4) selected for subsequent experiments due to its better knockdown efficiency. *F*: cell counting kit-8 (CCK-8) assay evaluating the effect of DLL4 knockdown on proliferation ability of sh-NC and sh-DLL4 mVECs. *G* and *H*: scratch assay and tube formation assay assessing the effect of DLL4 knockdown on migration and tube formation ability of mVECs (scale bar = 200 μm). *I*: Western blot analysis examining the impact of DLL4 knockdown on migration, angiogenesis, and Notch signaling pathway activity in mVECs. All cell experiments were performed in triplicate; *P < 0.05, **P < 0.01, ***P < 0.001. oe-DLL4, DLL4 overexpression; oe-NC, control cell overexpression.

**Figure 4.**
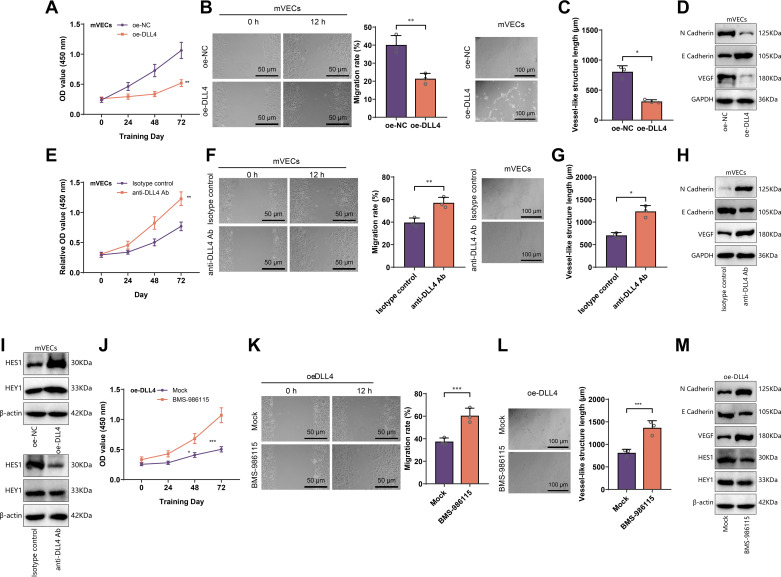
Impact of delta-like ligand 4 (DLL4) on vascular formation capacity in microvascular endothelial cells (mVECs). *A*: cell counting kit-8 (CCK-8) assay evaluating the effect of DLL4 overexpression (oe-NC and oe-DLL4) on the proliferation ability of mVECs. *B* and *C*: scratch assay and tube formation assay examining the influence of DLL4 overexpression on the migration and tube formation capacity of mVECs, with representative images on the left and quantification results on the right. *D*: Western blot analysis of the expression levels of migration and vascular formation-related proteins in each group. *E*–*H*: effect of anti-DLL4 antibody treatment on the proliferation, migration, and tube formation of mVECs. *I*: impact of DLL4 overexpression or anti-DLL4 antibody treatment on the expression of proteins involved in the Notch signaling pathway, as determined by Western blot. *J*–*M*: effect of the Notch inhibitor, BMS-986115, on the proliferation, migration, and tube formation of oe-DLL4 mVECs; scale bar = 200 μm. Cell experiments were repeated three times; *P < 0.05, **P < 0.01, ***P < 0.001.

To investigate the relationship between DLL4 expression and angiogenesis, we isolated and identified mVECs (Fig. 3*A*). Based on this, we established DLL4 overexpression and control cell lines (oe-DLL4 and oe-NC) (Fig. 3, *B* and *C*). The angiogenic capability of the cell lines was evaluated using proliferation, migration, and tube formation assays. The results showed that compared with oe-NC, oe-DLL4 exhibited significantly reduced proliferation, migration, and tube formation abilities (Fig. 4, *A–D*).

Next, we examined whether inhibiting DLL4 could enhance the angiogenic capability of mVECs. We generated DLL4 knockdown cell lines (sh-DLL4 and sh-NC) using mVECs as a basis (Fig. 3, *D* and *E*) and blocked DLL4 using anti-DLL4 antibody or its isotype control. Changes in various indicators of the cell lines were then evaluated. The results demonstrate a significant upregulation in proliferation, migration, and lumen formation capabilities in the sh-DLL4 or anti-DLL4 antibody group compared with the sh-NC or isotype control groups (Fig. 4, *E–H* and Fig. 3, *F–I*), suggesting that inhibiting DLL4 can promote the angiogenic capability of mVECs.

DLL4 is a cell membrane ligand that inhibits angiogenesis by activating downstream signaling pathways via Notch binding (24). To assess the activation of a Notch signaling pathway in each cell line, we examined the expression of downstream effectors HES1 and HEY1 (48). Western blot analysis revealed that HES1 and HEY1 expression in oe-DLL4 was significantly higher than in oe-NC, whereas sh-DLL4 and anti-DLL4 antibody groups exhibited significantly decreased expression compared with their respective controls (Figs. 4*I* and 3*I*), indicating that DLL4 expression positively regulates Notch signaling activation. Furthermore, we treated oe-DLL4 with BMS-986115 (a Notch inhibitor) and assessed changes in its angiogenic capability. The results demonstrated that BMS-986115 could reverse the inhibitory effects of DLL4 overexpression on mVECs’ proliferation, migration, and tube formation (Fig. 4, *J*–*M*), suggesting that the inhibitory role of DLL4 on the angiogenic capability of mVECs depends on the activation of the Notch signaling pathway.

Taken together, these findings indicate that inhibiting DLL4 promotes the proliferation, migration, and tube formation capabilities of vascular endothelial cells by suppressing the Notch signaling pathway.

### Enhanced Graft Survival in AFG by Inhibition of DLL4

**Figure 5.**
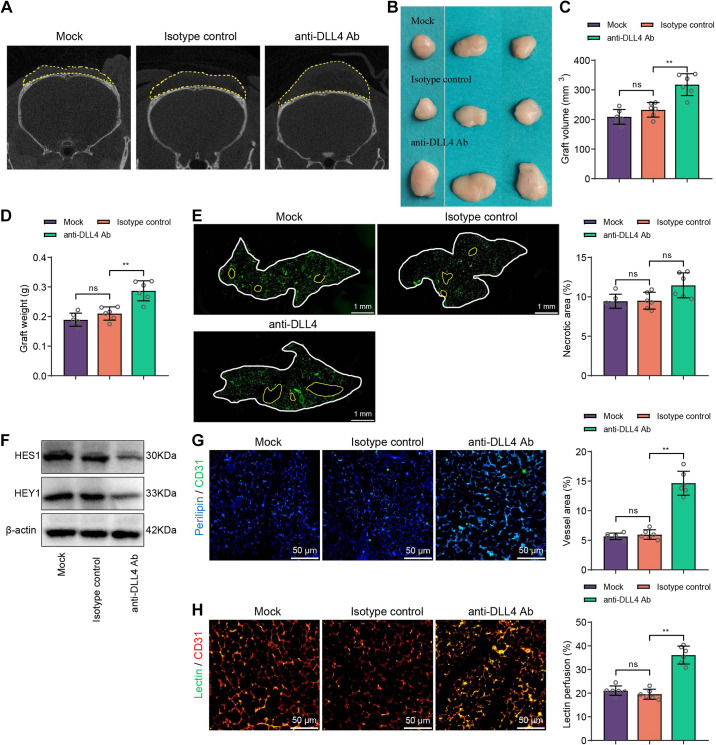
The impact of delta-like ligand 4 (DLL4) inhibition on the survival of autologous fat graft (AFG) mice. *A*: representative micro-computed tomography (micro-CT) images showing the survival of AFG mice in the Mock, isotype control, and anti-DLL4 Ab treatment groups after 12 wk of adipose tissue transplantation. The yellow dashed line indicates the area of the transplanted fat, with larger areas, indicating better survival outcomes. *B*: presentation of the transplanted tissues in each group. *C* and *D*: statistical results of volume and weight of the transplanted tissues in each group. *E*: immunofluorescence staining of adipocyte markers in the transplanted tissue of each group, with representative images on the left. The white dashed line indicates the area of the transplanted tissue, and the yellow dashed line indicates the area of necrotic tissue. Quantification of the necrotic area is shown on the right (scale bar = 2 mm). *F*: Western blot analysis of protein expression in the transplanted tissues of each group. *G* and *H*: immunofluorescence staining and lectin perfusion experiment examining the distribution and perfusion of blood vessels in the transplanted tissues (scale bar = 100 μm). Each group had a sample size of 6; ^ns^*P* > 0.05, **P < 0.01.

Based on in vitro experimental validation, we investigated the impact of inhibiting DLL4 on the survival of adipose-derived stem cells (ASCs) in vivo. As described in materials and methods, we established a mouse model of ASC transplantation and treated the mice with anti-DLL4 antibody (Ab) or its control (isotype control). After 12 wk posttransplantation, adipose tissue was harvested and examined. We observed that compared with the Isotype control treatment, the anti-DLL4 Ab-treated group exhibited increased graft volume and weight (Fig. 5, *A*–*D*), indicating improved survival of ASCs with anti-DLL4 Ab treatment. However, histological staining results showed no significant effect of anti-DLL4 Ab treatment on tissue necrosis (Fig. 5*E*).

Furthermore, we investigated the activation of Notch signaling and vascular distribution within the ASC tissue. We found that compared with the isotype control treatment, the anti-DLL4 Ab-treated group showed significantly reduced expression of HES1 and HEY1 (Fig. 5*F*) and increased vascular density (Fig. 5*G*). In addition, the vascular perfusion assay demonstrated a significant increase in the proportion of Lectin-positive vessels with anti-DLL4 Ab treatment (Fig. 5*H*), suggesting that anti-DLL4 Ab treatment not only promotes vascular quantity within the ASC tissue but also increases the proportion of functional vessels.

Taken together, these results indicate that DLL4 inhibition can promote the survival of ASCs in mice by enhancing vascular neoangiogenesis.

### Construction and Characterization of ADSC EVs-siDLL4

EVs are vesicular structures with a diameter of 30–150 nm that play a crucial role in numerous diseases (49). EVs serve as excellent bio-nanocarriers with various advantages (50). Previous studies have demonstrated the therapeutic potential of EVs in disease treatment through the delivery of siRNA (51). Moreover, it has been shown that ADSCs-derived EVs can enhance the survival rate of transplants during adipose tissue transplantation (47). Therefore, this study aims to investigate the potential application of EVs derived from ADSCs, loaded with and delivering siDLL4, in promoting angiogenesis and thus improving the survival of adipose fat grafts.

**Figure 6.**
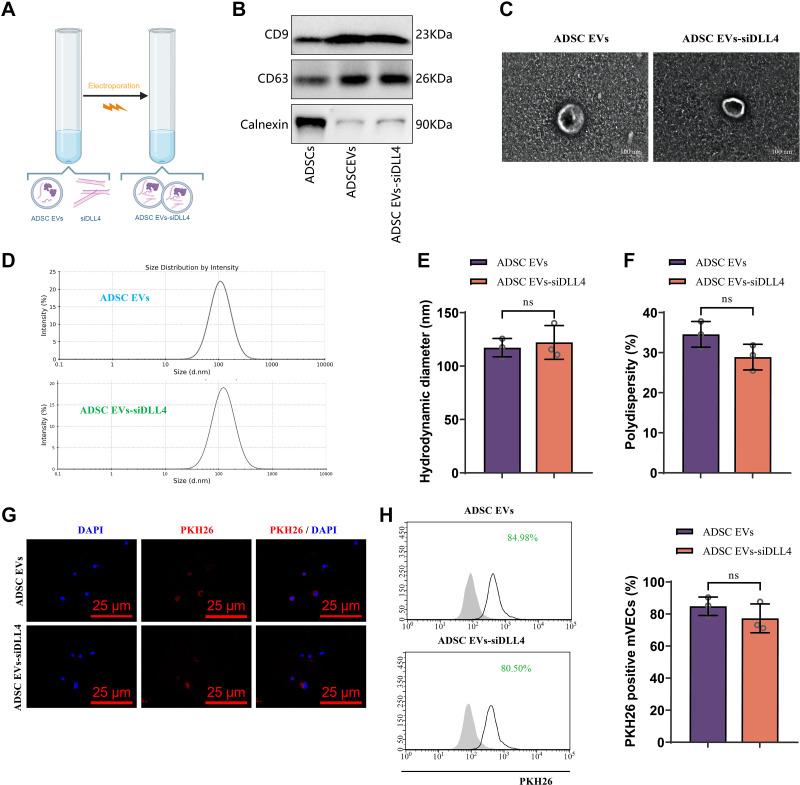
Characterization of adipose-derived mesenchymal stem cell (ADSC) and their extracellular vesicles (EVs) and ADSC EVs to carry DLL4 siRNA (ADSC EVs-siDLL4). *A*: schematic representation of ADSC EVs loaded with siDLL4 using electroporation. *B*: Western blot analysis of EVs-associated proteins in ADSC EVs and ADSC EVs-siDLL4, with ADSCs as a control. *C*: transmission electron microscopy images of ADSC EVs and ADSC EVs-siDLL4 (scale bar = 100 nm). *D*: size distribution of ADSC EVs and ADSC EVs-siDLL4 as measured by nanoparticle tracking analysis (NTA). *E* and *F*: comparison of size and percentage polydispersity index of ADSC EVs and ADSC EVs-siDLL4. *G* and *H*: fluorescence staining and flow cytometry analysis of mVECs uptake of ADSC EVs and ADSC EVs-siDLL4 (scale bar = 20 μm). Cell experiments were repeated three times; ^ns^*P* > 0.05. Created with BioRender.com.

**Figure 7.**
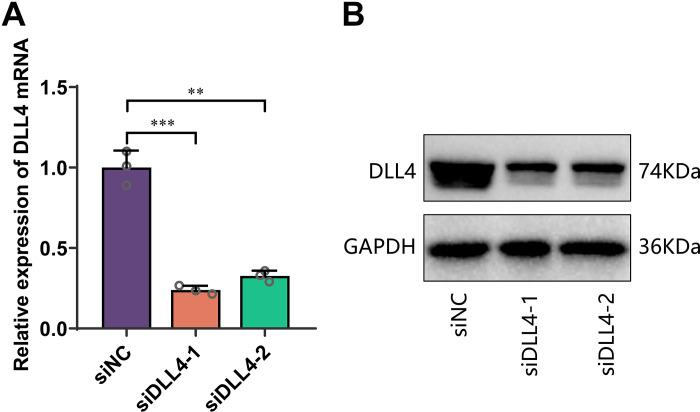
Validation of delta-like ligand 4 (DLL4) siRNA (siDLL4) knockdown efficiency. *A* and *B*: the knockdown efficiency of siDLL4 was examined by RT-qPCR and Western blot analysis. siDLL4-1 was selected as the most effective siRNA for subsequent experiments (referred to as siDLL4). Cell experiments were repeated three times; **P < 0.01, ***P < 0.001.

As described in materials and methods, EVs were isolated from ADSCs and loaded with siDLL4 using electroporation (Fig. 6*A*) (52). The knockdown effect of siDLL4 was validated through in vitro experiments before loading (Fig. 7, *A* and *B*). Characterization of ADSC EVs and ADSC EVs-siDLL4 was performed. Western blot analysis showed the expression of EV markers CD9 and CD63 in both ADSC EVs and ADSC EVs-siDLL4, whereas calnexin expression was negative (Fig. 6*B*). Transmission electron microscopy revealed that both ADSC EVs and ADSC EVs-siDLL4 exhibited cup-shaped or saucer-like vesicular structures, consistent with the basic characteristics of EVs (Fig. 6*C*). Nanoparticle tracking analysis (NTA) was used to evaluate size and concentration, demonstrating similar concentration curves for ADSC EVs and ADSC EVs-siDLL4 (Fig. 6*D*), and no significant difference in their mean diameter (Fig. 6*E*). The percentage dispersity index was also assessed for both ADSC EVs and ADSC EVs-siDLL4, indicating no significant difference in size uniformity between the two groups (Fig. 6*F*).

Finally, as described in materials and methods, the uptake of ADSC EVs and ADSC EVs-siDLL4 by mVECs was evaluated. EVs were labeled with PKH26 and coincubated with mVECs, followed by detection of uptake after 4 h. Immunofluorescence and flow cytometric analysis revealed that both ADSC EVs and ADSC EVs-siDLL4 were effectively taken up by mVECs, with no significant difference in the percentage of uptake between the two groups (Fig. 6, *G* and *H*).

Taken together, these results demonstrate the successful construction of ADSC EVs-siDLL4, which shares similar characteristics to ADSC EVs.

### Enhanced Suppression of DLL4-Notch Signaling and Improved Angiogenic Potential by ADSC EVs-siDLL4

We conducted in vitro validation of the effects of ADSC EVs-siDLL4. We treated mVECs with ADSC EVs-siDLL4 and established siNC (liposome-mediated), siDLL4 (liposome-mediated), and ADSC EVs treatment groups as controls. Subsequently, we examined the relevant indicators of each group.

**Figure 8.**
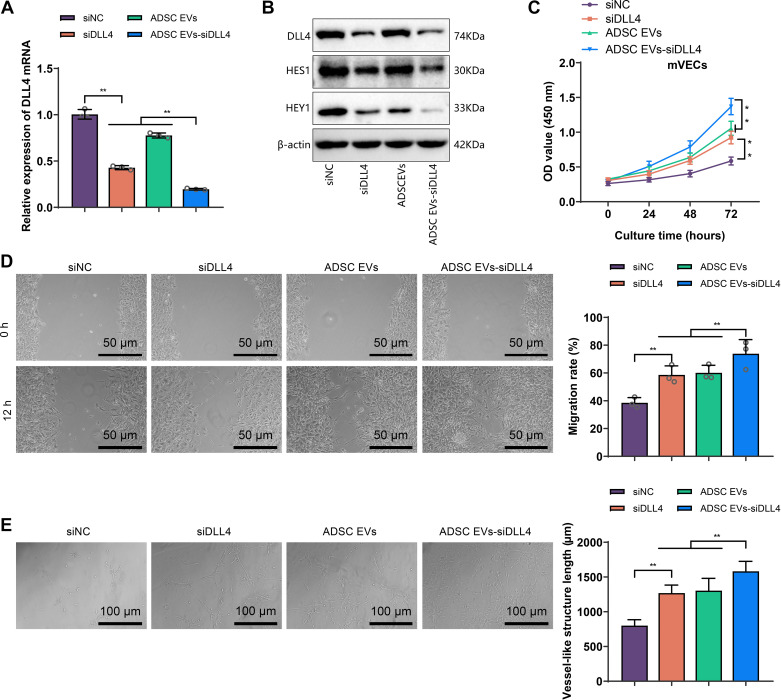
The effect of adipose-derived mesenchymal stem cell (ADSC) and their extracellular vesicles (EVs)-delta-like ligand 4 (DLL4) siRNA (siDLL4) on the angiogenic capacity of microvascular endothelial cells (mVECs). *A*: RT-qPCR analysis of the impact of siRNA negative control (siNC), siRNA for delta-like ligand 4 (siDLL4), ADSC EVs, and ADSC EVs-siDLL4 treatments on DLL4 mRNA expression in mVECs. *B*: Western blot analysis of the effect of siNC, siDLL4, ADSC EVs, and ADSC EVs-siDLL4 treatments on relevant protein expression in mVECs. *C*: cell counting kit-8 (CCK-8) assay to evaluate the proliferation ability of mVECs in each treatment group. *D* and *E*: evaluation of the migration and tube formation capacity of mVECs in the different treatment groups. Cell experiments were repeated three times; scale bar = 200 μm; **P < 0.01.

The results of the analysis showed that the level of DLL4 in the ADSC EVs-siDLL4 group was significantly lower than in the siDLL4 and ADSC EVs treatment groups (Fig. 8, *A* and *B*). This indicates that ADSC EVs-siDLL4 has a higher efficiency compared with liposome-mediated siDLL4 transfection. We also assessed the activation of the Notch signaling pathway in mVECs of each group. The Western blot results showed that similar to the trend of DLL4 expression, the Notch signaling activity was the lowest in the ADSC EVs-siDLL4 group (Fig. 8*B*).

Subsequently, we examined the impact of ADSC EVs-siDLL4 on the angiogenic ability of mVECs. The CCK-8 experiment results demonstrated that the cell proliferation ability was strongest in the ADSC EVs-siDLL4 treatment group (Fig. 8*C*). Similarly, the treatment with ADSC EVs-siDLL4 significantly enhanced the migration and tube formation ability of mVECs (Fig. 8, *D* and *E*).

These results indicate that compared with siDLL4 delivered by liposomes, ADSC EVs-siDLL4 can better suppress the expression of DLL4 and activate the Notch signaling pathway in mVECs. Furthermore, ADSC EVs-siDLL4 has a more pronounced promoting effect on the angiogenic ability of mVECs compared with siDLL4 alone or ADSC EVs.

### Enhanced AFG Survival and Vascularization by ADSC EVs-siDLL4

To investigate the impact of ADSC EVs-siDLL4 on the survival of ADSCs in vivo, we transplanted a mixture of ADSC EVs-siDLL4 and lipids. We also established treatment groups with siNC, siDLL4, and ADSC EVs (siNC and siDLL4 loaded in liposomes). After transplantation, we harvested adipose tissue at 12 wk and conducted examinations.

**Figure 9.**
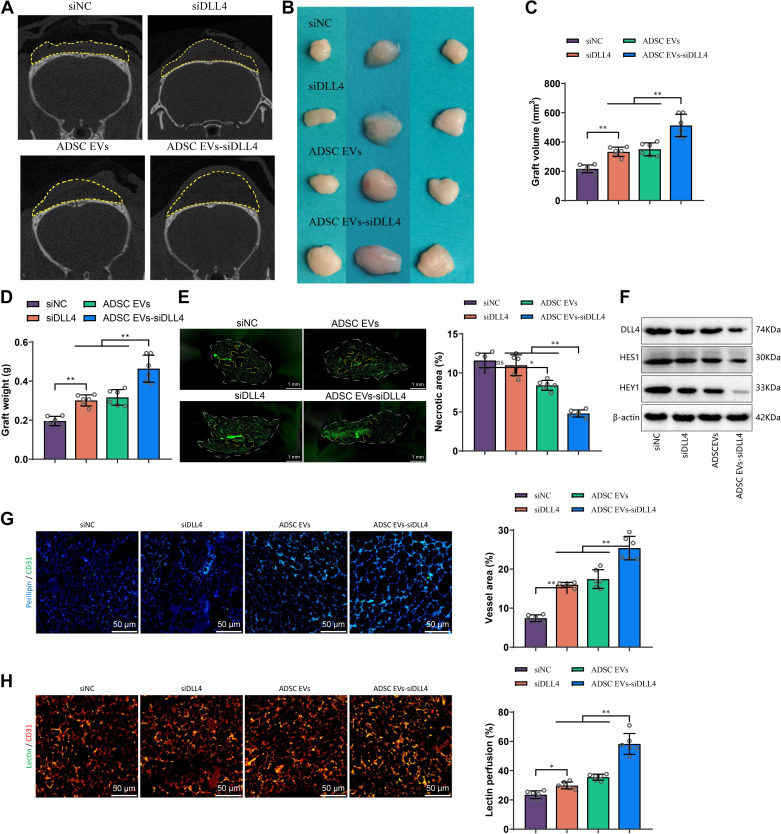
Effects of adipose-derived mesenchymal stem cell (ADSC) and their extracellular vesicles (EVs)-delta-like ligand 4 (DLL4) siRNA (siDLL4) on the survival of autologous fat graft (AFG). *A*: representative images of the survival of AFG in siRNA negative control (siNC), siRNA for delta-like ligand 4 (siDLL4), ADSC EVs, and ADSC EVs-siDLL4 treatment groups at 12 wk, with the yellow dashed lines representing the grafts and larger areas indicating better survival outcomes. *B*: display of the grafts in each treatment group. *C* and *D*: statistical results of graft volume and weight in each group. *E*: immunofluorescence staining for adipocyte markers in graft tissue, with representative images on the left side, the white dashed lines indicating the graft area, the yellow dashed lines representing the necrotic tissue area, and quantification of the necrotic area on the right side (scale bar = 2 mm). *F*: Western blot analysis of DLL4 and Notch signaling proteins in the tissue of each group. *G* and *H*: immunofluorescence staining and lectin perfusion experiments to evaluate vascular distribution and perfusion in graft tissue (scale bar = 100 μm). Each group had *n* = 6; ^ns^*P* > 0.05), *P < 0.05, **P < 0.01.

Consistent with the results of in vitro experiments, the ADSC EVs-siDLL4 group showed the best survival rate of AFG cells in mice (Fig. 9, *A*–*E*). Specifically, compared with siDLL4 and ADSC EVs alone, the ADSC EVs-siDLL4 group exhibited significant increases in transplanted volume and weight (Fig. 9, *A*–*D*). In addition, ADSC EVs-siDLL4 treatment led to substantial improvement in tissue necrosis (Fig. 9*E*), which may be attributed to the presence of biologically active substances in ADSC EVs.

Subsequently, we examined the expression of DLL4 and Notch signaling response proteins in AFG tissue. The results demonstrated that, compared with the siDLL4 and ADSC EVs treatment groups, ADSC EVs-siDLL4 significantly suppressed the expression of DLL4 and activation of the Notch signaling pathway (Fig. 9*F*). Furthermore, analysis of vascular distribution revealed that, compared with siDLL4 and ADSC EVs, the ADSC EVs-siDLL4 group exhibited a significant increase in both the degree of vascular abundance and the proportion of Lectin-positive vessels (Fig. 9, *G* and *H*).

Taken together, these findings suggest that ADSC EVs-siDLL4 can enhance vascular development and promote AFG cell survival.

## DISCUSSION

AFG is a widely used technique in plastic and reconstructive surgery, which involves the redistribution of autologous adipose tissue to improve tissue volume and shape (53, 54). However, the survival rate of transplanted fat cells in the new site is often low, which limits the effectiveness of the procedure (55, 56). To address this issue, numerous studies have explored the mechanisms and factors that promote AFG survival, including the role of angiogenesis (57, 58). However, to date, research on the mechanisms of AFG survival and angiogenesis remains relatively limited (57, 59, 60).

In this study, high-throughput transcriptome sequencing was used to identify differentially expressed genes associated with AFG survival. Compared with previous studies, we investigated a broader range of gene expression changes and discovered several novel potential regulatory factors. These differentially expressed genes included some nipple-related structural genes and angiogenesis-related genes previously reported in the literature (61–63). In addition, we identified new potential gene regulatory networks associated with AFG survival.

DLL4 is a significant regulator of angiogenesis that has been shown to play a crucial role in arterialization (64, 65). Our study found a significant increase in DLL4 expression levels in AFG tissue, which positively correlated with the survival rate of fat cells. Further experimental results demonstrated that DLL4 promotes angiogenesis, improves tissue perfusion, and consequently enhances AFG survival. These findings are consistent with previous research and provide new insights into the mechanisms underlying AFG survival.

To further investigate the mechanistic role of DLL4 in angiogenesis, we conducted overexpression and knockdown experiments of DLL4 in vascular endothelial cells. The results revealed that knockdown of DLL4 increased the proliferation, migration, and lumen formation capacity of mVECs, whereas overexpression of DLL4 led to a decrease in these capabilities. These findings align with previous studies and further underscore the crucial role of DLL4 in angiogenesis.

Based on the cumulative findings, we infer that downregulation of DLL4 enhances the survival rate of AFG by promoting angiogenesis. Our study corroborates existing research but offers a more detailed mechanistic explanation. By inhibiting DLL4 function, we can improve the survival rate of AFG and further optimize the outcomes of reconstructive and repair surgeries.

Furthermore, we explored the use of ADSC EVs as carriers for delivering DLL4 siRNA. In vitro experiments confirmed the efficacy of this approach, showing that siDLL4 within ADSCEVs could enhance the proliferation, migration, and lumen formation capability of mVECs. In addition, further experimentation demonstrated that using ADSC EVs loaded with siDLL4 significantly promote the survival rate of AFG. These results indicate the substantial potential of ADSC EVs as carriers for siDLL4, serving as a novel factor in promoting AFG.

We further explored the method of delivering DLL4 siRNA using ADSC EVs as carriers. In vitro experiments confirmed the effectiveness of this method and revealed that siDLL4 in ADSC EVs could inhibit the proliferation, migration, and lumen formation ability of mVECs. Furthermore, further experiments demonstrated that using ADSC EVs to deliver siDLL4 significantly promoted the survival rate of AFG. These results suggest that ADSC EVs, as carriers for siDLL4, have great potential as a novel AFG-promoting factor.

In conclusion, inhibiting DLL4 in vascular endothelial cells can increase the survival rate of AFG by promoting angiogenesis. ADSC EVs-siDLL4 has great potential in promoting AFG survival. This finding holds significant scientific significance and clinical value as it provides a new strategy and approach for improving AFG outcomes. The scientific significance of this study lies in its first-time revelation of DLL4 expression in AFG tissue and its discovery of its relationship with angiogenesis. DLL4 is mainly located in vascular endothelial cells and inhibits their proliferation, migration, and lumen formation ability through the activation of the Notch signaling pathway. Furthermore, further experimental results demonstrate that inhibiting DLL4 can increase AFG survival by promoting angiogenesis. The clinical significance of this study lies in providing a potential method for promoting AFG survival. Currently, the survival rate of AFG still has certain limitations. By inhibiting DLL4, we can promote angiogenesis, improve the blood supply status of AFG, and thereby increase its survival rate. In addition, the research team used ADSC EVs as lipid nanoparticle carriers, successfully delivering siDLL4 to cells and validating its roles in promoting angiogenesis and AFG survival. This research provides new insights for the development of novel AFG promoters and treatment strategies.

However, there are still some limitations in this study. First, the research results were obtained in a mouse model, so further animal experiments and clinical trials are needed before clinical application. Second, this study only focuses on the role of DLL4, while there may be other factors that play a significant role in AFG survival, which require further investigation. Future research can further explore other factors that promote angiogenesis and AFG survival and search for more effective carriers and delivery systems to deliver these factors. In addition, the findings of this study can be combined with other relevant research to improve the survival rate and clinical outcomes of AFG further. In the future, we may also explore the contents of EVs derived from ADSCs. Although our current research is primarily focused on delivering DLL4 siRNA through these EVs, analyzing their contents could potentially offer further insights for the transplantation process.

In summary, this study used RNA-seq technology to screen differentially expressed genes related to AFG survival and further explored the mechanism of DLL4 in angiogenesis. Our study found that DLL4 can increase the survival rate of AFG by promoting angiogenesis. Furthermore, we successfully delivered siDLL4 with the use of ADSC EVs as carriers and further verified its role in promoting AFG survival. These research results hold significant scientific significance and clinical value, providing new insights and methods for further optimizing AFG surgeries. However, we need to be mindful of the limitations of this study in clinical application and explore more AFG-promoting factors and strategies to meet the needs of different patients.

## ETHICAL APPROVALS

All experiments involving mice were approved by the Animal Ethics Committee of Chongqing Hospital of Traditional Chinese Medicine.

## DATA AVAILABILITY

All data can be provided as needed. RNA seq data can be obtained by contacting the corresponding author.

## GRANTS

This study was supported by Chongqing Banan District Science and Health Joint Medical Research Project under Grant No. BNWJ202300128.

## DISCLOSURES

No conflicts of interest, financial or otherwise, are declared by the authors.

## AUTHOR CONTRIBUTIONS

S.-L.D., Q.F., Q.L., F.-J.H., and M.Z. conceived and designed research; S.-L.D. and X.Z. drafted manuscript; X.Z. approved final version of manuscript.
